# Targeting ADAM10 in Cancer and Autoimmunity

**DOI:** 10.3389/fimmu.2020.00499

**Published:** 2020-03-24

**Authors:** Timothy M. Smith, Anuj Tharakan, Rebecca K. Martin

**Affiliations:** Department of Microbiology and Immunology, School of Medicine, Virginia Commonwealth University, Richmond, VA, United States

**Keywords:** metalloproteases, ADAM10 inhibitors, NKG2D, hodgkin lymphoma, glioblastoma, breast cancer, systemic lupus erythematosus, rheumatoid arthritis

## Abstract

Generating inhibitors for A Disintegrin And Metalloproteinase 10 (ADAM10), a zinc-dependent protease, was heavily invested in by the pharmaceutical industry starting over 20 years ago. There has been much enthusiasm in basic research for these inhibitors, with a multitude of studies generating significant data, yet the clinical trials have not replicated the same results. ADAM10 is ubiquitously expressed and cleaves many important substrates such as Notch, PD-L1, EGFR/HER ligands, ICOS-L, TACI, and the “stress related molecules” MIC-A, MIC-B and ULBPs. This review goes through the most recent pre-clinical data with inhibitors as well as clinical data supporting the use of ADAM10 inhibitor use in cancer and autoimmunity. It additionally addresses how ADAM10 inhibitor therapy can be improved and if inhibitor therapy can be paired with other drug treatments to maximize effectiveness in various disease states. Finally, it examines the ADAM10 substrates that are important to each disease state and if any of these substrates or ADAM10 itself is a potential biomarker for disease.

## Introduction

A Disintegrin And Metalloproteinase (ADAMs) are type I transmembrane endopeptidases that are a member of the metzincin superfamily which share a zinc-binding consensus motif that is required for catalytic activity. The metzincin superfamily includes ADAMs along with matrix metalloproteases (MMP), and ADAM-thrombospondins (ADAM-TS). ADAM10 is a modular protein comprised of metalloprotease, cysteine-rich, disintegrin, and epidermal growth factor (EGF)-like domains (1, 2). While all functionally active metzincin proteases contain a zinc binding motif, ADAMs are unique in that they also contain a transmembrane domain and are active while membrane bound. ADAM are membrane-anchored and cleave ligands expressed on the surface through a process known as ectodomain shedding. ADAMs also mediate regulated intramembrane proteolysis (RIP) of transmembrane proteins. Following translation of ADAM10 mRNA, the prodomain is cleaved by Furin (3, 4). With the removal of the prodomain, the disintegrin and cysteine rich domains engulf the active metalloproteinase domain. This is thought to be autoinhibitory to add a layer of control to the mature protease (5). Once ADAM10 leaves the endoplasmic reticulum, it can directly associate with all members of a subgroup of tetraspanins, Tspan8, all of which contain eight cysteines in the large extracellular domain (6). These tetraspanins can alter the substrate-specify of ADAM10 through conformational change (6). Synapse-associated protein 97 (SAP97) trafficks ADAM10 to the golgi apparatus (7). ADAM10 either interacts with its substrates at the cell surface or, as with L1, CD44, or CD23, ADAM10 meets these substrates in the endosomal pathway, leading to possible packaging into exosomes (8).

It is now recognized that there are 38 members of the ADAM family that are conserved between invertebrate and vertebrate evolution, with humans harboring 13 proteolytically active ADAMS (9–13). Of all the ADAMs, ADAM17 is the most similar to ADAM10 with regard to structure and function. According to the National Center for Biotechnology Information (NCBI) database, human ADAM10 RNA-seq data from 95 individuals from 27 different tissues showed ubiquitous expression amongst all 27 tissues (14). ADAM10 might be best known historically for its role in Notch signaling, and more recently for cleaving the amyloid precursor protein (APP) associated with the pathophysiology of Alzheimer's disease. It is important to note, however, ADAM10 has over 40 other substrates and counting that are involved in a multitude of biological functions including inflammation, apoptosis, cell adhesion, cell metabolism, cancer proliferation, cancer metastasis, and autoimmunity in addition to other functions [reviewed in (15, 16)]. ADAM10 is crucial for development as mice that are deficient in ADAM10 die around day 9.5 during the early embryonic stages (17). Some of the characterized ligands for ADAM10 in addition to APP and Notch are: E-cadherin, L-selectin, EGF, FASL, CD40L, ligand for inducible T cell costimulatory (ICOS-L), MICA, MICB, and ULBP2 (18–25).

## ADAM10, Notch, and the Immune System

ADAM10's ability to regulate Notch signaling has been well-characterized and extensively reviewed (16, 26–29), including a recent review by Lambrecht et al. discussing the role of ADAMs in the immune system (30). Briefly, Notch signaling regulates many different important processes in cellular differentiation, including the development and differentiation of both innate and adaptive immune cells. Generally, the initial cleavage of Notch by furin-like convertase at s1 generates the mature Notch protein as it is transported to the cell membrane (29). Notch engagement with a Notch ligand initiates ADAM10 mediated proteolysis of the extracellular domain of the Notch receptor at the s2 cleavage site. The extracellular domain is released and endocytosed by the adjacent ligand-expressing cells. This cleavage event, produces a substrate that can be cleaved by the γ-secretase complex (S3 cleavage) (31, 32). S3 cleavage releases the Notch intracellular domain (NICD), this translocates to the nucleus where it complexes with transcription factor RBP-Jκ to induce transcription of Notch target genes (31, 33). In addition to ADAM10's importance in Notch signaling, some of ADAM10's most well-characterized substrates are also Notch receptors, such as Delta-like 1–4 and Jagged 1–2 (34).

The loss of ADAM10 in B cell development results in the loss of the marginal zone B cell (MZB) compartment (35). This has been shown to be mediated through Notch2 activation. Overexpression of ADAM10 in hematopoiesis results in the complete loss of the B cell compartment and an overall myeloid expansion that is Notch dependent (32). For B cells, ADAM10 substrates that regulate cell activation and antibody production include but are not limited to, Notch, ICOSL, and CD23 (23, 35, 36).

For T-cells, the strength of Notch signaling has been implicated in both the lineage decision between CD4^+^ and CD8^+^ T-cell subsets as well as between αβ and γδ T cell subsets (37–39). In addition, Notch 1 activation through binding of various Notch ligands on antigen presenting cells, such as dendritic cells, skew CD4 cells toward T-helper subsets, induce T-cell proliferation, and control survival of CD4^+^ memory cells (40, 41). Additional ADAM10 substrates control a wide variety of processes. These include CD44, which alters T cell migration (42, 43), FAS ligand (FASL), which in a soluble form acts as a decoy receptor to reduce activation induced apoptosis (44), and persistent Lymphocyte Activating 3 (LAG3) or T cell immunoglobulin and mucin domain-containing protein 3 (TIM3), which are markers of T cell exhaustion in tumor-infiltrating lymphocytes (45).

ADAM10 is ubiquitously expressed in human tissue with a vast array of substrates. Dysregulation or inhibition of ADAM10 can affect or result in the pathophysiology of a wide range of diseases. This review will focus on characterizing ADAM10 and the potential use of ADAM10 inhibitors in the context of cancer and autoimmunity.

## ADAM10 in Cancer

### Glioblastoma

Glioblastoma (GBM) is the most aggressive form of glioma resultant from malignant astrocytes in the brain. The link between ADAM10 and GBM disease progression is demonstrated in many studies (46–49). In a study of 50 GBM patients, Kanaya et al. demonstrated that low ADAM10 expression levels in tumor specimens positively correlated with increased survival especially when paired with tumor resection, as opposed to high ADAM10 levels (50). Interestingly, no detectable ADAM10 expression was reported in normal brain tissue (50).

There are several proposed mechanisms of ADAM10's promotion of GBM. Neuroligin-3 (NLGN3) is released from neurons by ADAM10. In multiple recent reports, NLGN3 levels have been linked with high grade GBM (46, 47). In GBM patients NLGN3 levels are high in the deep brain, preparing a pro-GBM tumor microenvironment. This expression in the deep brain is not seen in normal brain tissue (46). NLGN3 acts on GBM, promoting proliferation through the P13K-mTOR pathway, pro-oncogenic gene expression through focal adhesion kinase (FAK), and synapse-related gene expression (46). Reportedly, patient-derived xenografts (PDX) would not grow in NLGN3 knockout mice. Using the ADAM10 inhibitors GI254023X and INCB7839 in mice, the growth of adult and pediatric glioblastoma cell lines, and PDX were inhibited (46, 47). These effects of ADAM10 inhibition were not directly on the tumor, but were shown to be mediated through the blockage of ADAM10 cleavage of NLGN3 from neurons (46, 47).

Another proposed mechanism implicates ADAM10's cleavage of N-cadherin in cell migration and metastasis (49). *In vitro* experiments where GBM cell lines were treated with an antibody to inhibit ADAM10 (Millipore #AB19026) found decreased tumor growth and migration. This was shown to be driven by cleavage of N-cadherin (49). Musumeci et al. examined 25 grade IV GBM specimens as compared to normal brain tissue controls to identify molecular markers of aggressiveness (48). ADAM10 protein and mRNA were positively correlated with GBM and surface N-cadherin protein was negatively correlated with GBM (48).

Natural killer cells (NKs) have been reported to have an anticancer immune response against GBM and are associated with improved prognosis (51). NK cells recognize GBM by binding ligands for the NKG2D receptor that are expressed in the malignant state. NKG2D binds to MICA, MICB, and ULBP1-6. MICA and ULBP2 are both cleaved by ADAM10 and ADAM17 (52, 53). Loss of NK cell activation of the NKG2D receptor allows GBM escape due to reduced activation of the NK cell cytotoxic effector state (24, 54). Using the ADAM10 specific inhibitor GI254023X, the dual ADAM10/ADAM17 inhibitor GW280264X, or siRNA inhibition of ADAM10 or ADAM17, Wolpert et al. demonstrated increased surface expression of ULBP2 in GBM stem cell lines (55). This subsequently increased the immunogenicity of these GBM stem cell lines (55).

In GBM, M2 macrophages are correlated with poor prognosis. Gjorgjevski et al. examined tissue from 20 GBM and using qRT-PCR of M1/M2 related genes to various protease genes, including *ADAM10* (56). A positive correlation was established between *ADAM10* expression and M1-related genes (56). This overall signature then positively correlated with better prognosis. Although this disagrees with the majority of the work done on GBM and ADAM10, the authors attributed the increased survival to the M1-skewed profile (56).

Overall, in GBM, ADAM10 has strong value as a biomarker for prognostic use. A large scale study is warranted to validate ADAM10 as predictive biomarker. ADAM10 appears to be a strong therapeutic candidate to target GBM due to the multiple substrates it cleaves that are implicated in disease progression. Even with very strong pre-clinical evidence, there has yet to be a clinical trial in GBM with ADAM10 inhibitors. This is most likely due to the failures that the ADAM10 inhibitors have been in clinical trials (57). Despite this, the use of ADAM10 inhibitors as a clinical intervention should be carefully evaluated due to ADAM10's role in the cleavage of amyloid plaque precursors (58, 59).

### Hodgkin Lymphoma, Non-hodgkin Lymphoma, and Multiple Myeloma

Hodgkin lymphoma (HL) is characterized by a clonal malignant lymphoproliferation in the form of lacunar histiocytes and Reed-Sternberg cells (60). Similar to GBM, ADAM10 promotes an immunosuppressive microenvironment through cleavage of the stress receptors MICB and the ULBP2, resulting in HL that has foregone immune surveillance (61, 62). Zocchi et al. generated two ADAM10 specific inhibitors (LT4 and MN8) (63). They found that treatment with either inhibitor blocked shedding of NKG2D-L in cultured HL samples and HL cell lines developed increased sensitivity to NKG2D-L-mediated killing after inhibitor treatment (63). Multiple studies have described the presence of ADAM10 in extracellular vesicles (EVs) released by the HL cells (64, 65). ADAM10 has additionally been described in EVs released from other tumors, including melanoma, GBM, lung, and colon cancer (66). In both HL studies, CD30 was found to be co-released on these HL EVs. This was proposed to further promotes an immunosuppressive tumor microenvironment (64). Interestingly, following treatment with the ADAM10 inhibitors (LT4 and CAM29), Tosetti et al. report that the inhibitor is additionally secreted in EVs leading to uptake by bystander cells (64). Overall, ADAM10 inhibitor treatment results in the restoration of membrane CD30 levels, which restored sensitivity to anti-CD30 monoclonal therapies used in HL, such as Iratumumab (64).

Non-hodgkin lymphoma (nHL) describes a variety of lymphomas, including Burkitt's lymphoma, diffuse large B cell lymphoma (DLBCL), and marginal zone lymphoma. All of these have in common the lack of Hodgkin cells. The prognosis for nHL can be worse due to the higher frequency of late-stage diagnoses (67). A variant of nHL is DLBCL. Epstein Barr-virus-positive (EBV^+)^ DLBCL, not otherwise specified (NOS) have been shown to have increased expression of the immunosuppressive molecule PD-L1 (68). PD-L1^+^ DLBCLs can be treated with anti-PD-L1 monoclonal therapy. However, some tumors fail to respond despite being PD-L1^+^. A correlative study using data from the cancer genome atlas found that DLBCLs with a low PD-L1 protein-to-mRNA ratio while also having higher relative expression levels of ADAM10 or ADAM17 had worse overall survival than high PD-L1 protein-to-mRNA counterparts (68). Two recent studies demonstrated that ADAM10 and ADAM17 can cleave PD-L1 in culture (69). With these data, the regulation of the PDL-1/PD-1 pathway can be better understood. Soluble PD-L1 is not completely understood, thus more work is needed to determine if inhibition of this part of the pathway will be helpful. Then we will know if ADAM inhibition will be helpful or harmful in nHL.

Multiple myeloma (MM) is characterized by a clonal expansion of plasma cells filling the bone marrow volume >30%. These cells often secrete large amounts of monoclonal Ig into the blood. This leads to a loss of structural integrity of the bone resulting in increased risk of bone fracture, as well as kidney damage due to the accumulation of monoclonal Ig deposits (70). In a study utilizing human MM cells, doxorubicin was used to model genotoxic stress. This upregulated ADAM10 and resulted in increased shedding of MICA and MICB. The increased ADAM10 expression in MM was associated with a senescent phenotype (71). This finding suggests that ADAM10 inhibitors may enhance NK cell immunotherapy in multiple myeloma and other cancers.

### Breast Cancer

**Figure 1:**
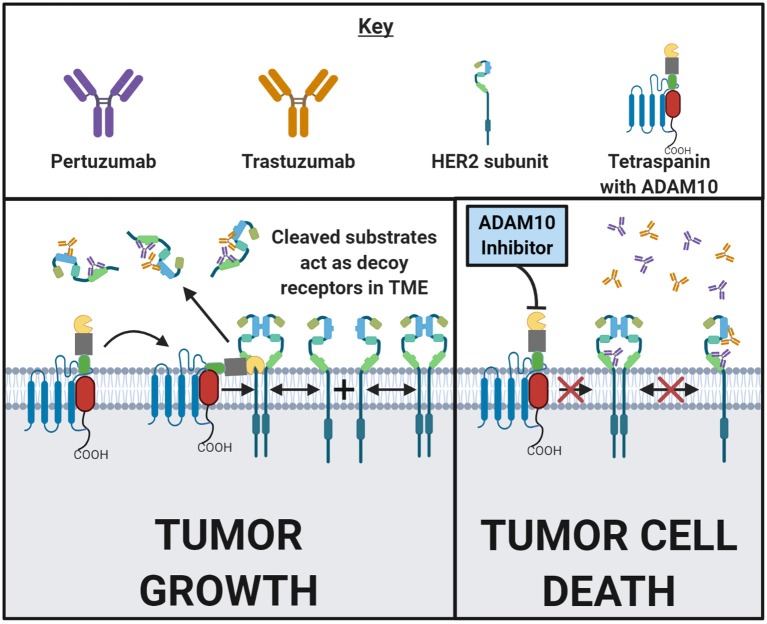
ADAM10 mediates cell surface cleavage of a large repertoire of substrates that can promote a pro-growth environment in malignant tumors. Some of these ADAM10 substrates, such as HER2, are targeted by FDA approved drugs. Following HER2 subunit dimerization with HER- family subunits, and upon ligand-binding or autoactivation, a pro-growth signal cascade is initiated which then drives malignancy. Trastuzumab and pertuzumab are both monoclonal antibodies that bind to two distinct epitopes of HER2 to prevent homodimerizaton and heterodimerization, respectively. In addition to inhibiting dimerization, tumor cells with bound antibody have an increased likelihood of succumbing to antibody dependent cellular cytotoxicity (ADCC) or opsonization. In the left and right panels above, a HER2-positive cancer cell surface in the presence of trastuzumab and pertuzumab is depicted. In the left panel, ADAM10 cleaves the extracellular domain of HER2 from the cell surface into the tumor microenvironment (TME). These cleaved domains then act as decoy receptors, decreasing the amount of trastuzumab or pertuzumab that binds to the tumor cell. The likelihood of ADCC or opsonization is now decreased. In the right panel, treatment with trastuzumab or pertuzumab is combined with the inhibition of ADAM10. Inhibiting ADAM10 results in less HER2 cleavage which reduces the amount of decoy receptors in the TME. Decreasing the decoy receptors in the TME and increasing the amount of HER2 on the tumor cell might enhance trastuzumab's and/or pertuzumab's antitumor effects. This general concept can be applied to other ADAM10 substrates in other disease states. Made in ©BioRender - biorender.com.

The human epidermal growth factor receptor (HER) family consists of four receptors: EGF receptor (EGFR), HER2, HER3, and HER4. HER2 overexpression occurs in ~10–20% of all invasive ductal mammary carcinomas of no special type (NST) and these are treated with trastuzumab, which is a monoclonal anti-HER2 antibody (72). While patients with HER2^+^ breast cancers often initially respond well to trastuzumab, it is common for cancer to relapse in a more resistant form (73). ADAM10 is known to cleave HER2. Increased shedding of HER2 into a soluble form, HER2-extra cellular domain (ECD) by ADAM10 has recently been shown to be predictive of reduced progression-free survival (74). In 545 HER2 positive invasive ductal mammary carcinoma (NST) patients, it was found that a high serum HER2-ECD relative to tumor HER2 level was predictive of reduced progression-free survival (74). Feldinger et al. demonstrated that after treatment with trastuzumab, ADAM10 levels increased both *in vitro* and *in vivo* using PDX (75). Treatment with the ADAM10 inhibitor INCB8765 increased sensitivity to trastuzumab *in vitro*, as well as restored responses in trastuzumab-resistant cells (75) (Model Figure 1). A different study confirmed these results in esophageal cancer PDX models and proposed that ADAM10 is conferring this resistance through the cleavage of the HER3 ligand (NRG-1β) (76). When HER2 is lost through trastuzumab treatment, HER3 is upregulated in a compensatory manner (76). The observed increase in ADAM10 then releases the ligand for HER3 and confers trastuzmab resistance. This can be reversed with ADAM10 inhibitor (GI254023X) treatment (76). Further studies are needed to confirm this mechanism in breast cancer as well as to examine the additive effects of ADAM10 inhibitors in combination with monoclonal anti-HER2 therapies, but the cleavage product HER2-ECD shows promise as a prognostic biomarker.

Triple negative breast cancer (TNBC) is a variant of breast cancer that exhibits little to no expression of the HER2, progesterone receptor (PR), or estrogen receptor (ER) and is often correlated with poor prognosis due to limited treatment options (77). Both ADAM10 and ADAM17 have been found to be expressed in the majority of TNBCs (78). In a study that used RNAi knockdown of ADAM10, or the ADAM10 inhibitor GI254023X in several different TNBC cell lines such as MDA-MB-231 and BT20, it was found that ADAM10 knockdown decreased *in vitro* cell migration (79). A different study that examined five TNBC cell lines reported finding that microRNA-365, (miR-365) directly interacts with the 3'-UTR of ADAM10 mRNA. Moreover, they reported that re-expression of ADAM10 led to the restoration of the cells ability to proliferate, migrate, and invade which was suppressed when overexpressing miR-365 (80). TNBCs have been reported to have the highest levels of PD-L1 amongst breast cancer types. Much like with DLBCLs, it has been proposed that ADAM10 may play a role in regulating the PD-1/PD-L1 axis (69). Recently, a study done on non-luminal breast cancers (Her2^+^ and TNBC), published in *EBioMedicine*, identified soluble APPα that is generated by ADAM10 cleavage of APP, as important in breast cancer tumor migration and proliferation (81). The importance of APP was shown using APP knockdown tumors *in vitro* and *in vivo*. This was also shown by knockdown of ADAM10 using RNAi (81). ADAM10 cleavage of APP in Alzheimer's disease has been extensively studied [and is reviewed in (82, 83)]. The expansion of importance in breast cancer emphasizes the examination of ADAM10 cleavage of APP in other cancers.

The xenoestrogens bisphenol-A (BPA) and nonylphenol (NP) are used by the plastics industry in products for human use (84). Urriola-Muñoz et al. demonstrated that both BPA and NP increased ADAM10 and ADAM17 activity, increasing the release of several EGFR ligands (84). Given the variety of important substrates of ADAM10 and ADAM17 in breast cancer as well as other cancers, this study sheds new light on the potential complications associated with these common xenoestrogens. Much more research will need to be done.

### Oral Squamous Cell Carcinoma

Oral squamous cell carcinoma (OSCC) is an invasive carcinoma derived from malignantly transformed squamous epithelium lining the oral cavity (85). Based on 80 cases of OSCC samples analyzed, it was determined that OSCC expressed high levels of ADAM10 and that the samples with the highest ADAM10 expression were found in metastatic lesions (86). Tissue inhibitor of metalloproteinase-3 (TIMP-3) has been shown to inhibit OSCC cell growth, angiogenesis, migration, and invasion (87). TIMP-3 is a secreted protein that binds to the ECM and inhibits metalloproteinase activity, with a particular affinity for suppression of ADAM10 activity (87). One study showed that in OSCC, increased hypermethylation of the TIMP-3 promotor led to reduced TIMP-3 mRNA expression. Treatment with a DNA methytransferase inhibitor (DNMTi) or overexpression of TIMP-3 reversed tumor cell migration, proliferation, and reduced the epithelial to mesenchymal transition (87). These effects may be due to loss of ADAM10 activity, but further work would need to be done to show this. Another recent study using HEK293 cells that overexpress TIMP-3 show evidence of reduced ADAM10-specific substrates after secretome analysis by mass spectrometry label free quantification. This study showed that with TIMP-3 overexpression, several ligands for the low-density-lipoprotein receptor-related protein-1 (LRP-1) are upregulated, such as macrophage inhibitor factor (MIF) (88). As TIMP-3 also binds to LRP-1, overexpression of TIMP-3 outcompetes other ligands of LRP-1 and thus the cell compensates by increasing expression (88). The authors of this study warn that the use of TIMP-3 interventions as an ADAM10 regulator could yield these alterations to the secretome and results might be unanticipated.

As in HL and GBM, OSCC are reported to have low expression levels of stress receptor, MICA, which has been shown to be under the regulation of ADAM10. In OSCC, the cleavage of MICA reduces tumor immunogenicity (89). Upon over-expressing MICA in the human squamous cell line SCC-25, increased NK cell killing was observed. This suggests ADAM10 regulation of stress receptors may also be occurring OSCC (89).

### ADAM10 and ADAM10 Substrates as Biomarkers in Cancer

A multi-center cross-validation study (1558 enrollment) was conducted to assess Heat shock protein 90α (Hsp90α) as a pan-cancer biomarker (90). A siRNA screen identified ADAM10 as responsible for the release of Hsp90α. This was validated through the use of the ADAM10 inhibitor (GI254023X) on tumor cell lines, where reduced release of Hsp90α was observed (90). The study identified ADAM10 as another potential biomarker in conjunction with Hsp90α, and looked at the pair as an exosomal biomarker (90). Overall, it emphasized the strength of Hsp90α, as a potential pan-cancer biomarker. Another study identified serum ADAM10 levels as a biomarker for disease in colorectal cancer by ELISA. This study also found ADAM10 had a minor, yet significant positive correlation with clinical stage (91). In sacral chordoma, a rare malignant primary bone tumor in the spine, a study spanning seven years positively correlated ADAM10 levels with increased metastasis, disease-free survival, overall survival, and histological type (92). Low ADAM10 expression by histochemical staining in patient tumors equated to longer survival as compared to high ADAM10 expression (92). Many different tumor types have identified ADAMs as potential biomarkers for disease or disease progression, the evidence for ADAM10 as biomarker is building and warrants larger-scale multi-center validation studies in order to be implemented in the clinic.

## ADAM10 in Autoimmunity

### Rheumatoid Arthritis

Rheumatoid arthritis (RA) is a chronic inflammatory disease affecting synovial joints, resulting in synovitis, cartilage destruction, and joint ankylosis. The pathophysiology of RA is largely driven by autoreactive antibodies directed against neoantigens generated by post-translational citrullination and carbamylation of self-peptides (93). These autoantibodies, which can be detected prior to the onset of clinically evident disease, form immune complexes which deposit in synovial joints and induce local inflammatory responses at articular surfaces (93). Given the role of ADAM10 in regulating antibody production and inflammatory responses, it is considered a promising therapeutic target to control RA disease activity and progression.

Cleavage of ADAM10 substrates in synovial tissue is involved in several pro-inflammatory processes. CXCL16, which requires ADAM10 cleavage to exert its biological effects, functions as a chemotactic signal for effector and memory T-helper 1 cells. ADAM10 and CXCL16 are upregulated in synovial joint biopsies from RA patients compared to healthy controls (94). These molecules are co-expressed on the surface of synovial macrophages, where they function to drive the accumulation of effector T-helper 1 cells, thereby promoting local inflammatory processes and exacerbating joint injury (94). *In vitro* siRNA knockdown of ADAM10 in RA-patient derived synovial fibroblasts also suppressed the release of the proinflammatory cytokines TNF-α, IL-6, and IL-8. These findings indicate that inhibition of ADAM10 may be effective in the treatment of RA by suppressing pro-inflammatory signaling within synovial tissue.

An early histological hallmark of RA pathogenesis is synovial angiogenesis, which permits leukocyte infiltration and progression to synovitis (93). Recent studies demonstrate that ADAM10 is upregulated in endothelial cells and synovial lining fibroblasts in RA tissue biopsies compared to osteoarthritis and healthy patients (95). Additionally, *in vitro* studies reveal that siRNA knockdown of ADAM10 impairs angiogenesis and suppresses VEGF release in endothelial cell lines (95, 96). Administration of this siRNA *in vivo* in a murine model of collagen-induced arthritis improved arthritis symptoms and reduced serum levels of the angiogenic cytokine VEGF (97). The involvement of ADAM10 in angiogenic processes in RA progression indicate that early inhibition of ADAM10 may slow or halt disease progression.

An additional, particularly debilitating outcome in RA is bone erosion (98). Erosion of bone in RA is thought to result from a local inflammatory milieu driving exaggerated osteoclast activity and invasion into periosteal regions (93). Endothelial cell ADAM10 has been shown to modulate osteoclast function. Murine models of ADAM10 deletion in endothelial cells led to reduced osteoclast numbers at the chondro-osseous junction and impaired long bone growth, indicating abnormal osteoclast function (99). Thus, inhibition of ADAM10 may restrain osteoclast activity and reduce the incidence of bone erosion in RA.

Though ADAM10 appears to be a promising therapeutic target in the management of RA progression, it may also provide a biomarker for predicting patient responsiveness to biologic therapies. A recent study demonstrated that elevated serum ADAM10 positively predicts treatment responsiveness to tocilizumab, a monoclonal antibody targeting the IL-6 receptor (100). Patients who were responsive to tocilizumab therapy had a roughly 6-fold higher baseline ADAM10 level compared to non-responders. Therefore, routine testing of RA patients for ADAM10 serum levels may offer guidance for the use of targeted therapies for specific patient groups in RA.

To date, the evidence regarding the role of ADAM10 in RA indicates that it would likely be a useful therapeutic target. Many of the existing small molecule inhibitors of ADAM10, such as GI254023X, INCB3619, and INCB7839, have the added benefit of inhibiting the ADAM10 homolog ADAM17. ADAM17 is a sheddase for a variety of pro-inflammatory molecules that are involved in RA pathogenesis (101–103). Thus, these drugs may provide a significant clinical benefit in the management of RA symptoms.

At present, none of the ADAM10 inhibitors have been evaluated for efficacy in clinical trials for RA. Clinical trials of GI254023X were discontinued in phase I/II due to hepatotoxicity following systemic administration (104). Preliminary studies of INCB7839, however, suggest that it is safe and well-tolerated for systemic use indicating that this class of drugs may have promise in the management of RA (105).

### Systemic Lupus Erythematosus

Systemic lupus erythematosus (SLE) is a common, multisystem autoimmune disorder that is characterized by aberrant antibody production directed against nuclear antigens such as double-stranded DNA and small nuclear ribonucleoproteins (106). These autoantibodies form immune complexes which deposit within tissues and promote organ dysfunction. Therefore, an effective therapeutic approach to SLE would require suppression of antibody production or the inflammatory reactions to immune complex deposition.

Numerous studies have demonstrated hyperactivity of the B cell activating factor (BAFF)—transmembrane activator and CAML interactor (TACI) system in SLE patients as well as in murine models of SLE (107, 108). Binding of BAFF by TACI on B cells causes excessive proliferation and T-independent activation of low-affinity self-reactive B cells. This activation leads to increased antibody production against harmless self-antigens which promotes disease progression in SLE (109). Recently, TACI was identified as a substrate for cleavage by ADAM10 (110). ADAM10-induced cleavage of TACI induces ectodomain shedding and the generation of soluble TACI (sTACI). This allows sTACI to function as a decoy receptor by binding soluble BAFF and APRIL to prevent T-independent B cell activation (110). These findings indicate that activation of ADAM10 in SLE may suppress aberrant B cell activity and reduce the synthesis of autoreactive antibodies.

ICOSL is another ADAM10 substrate that has been linked to abnormal antibody production (23). B cell ADAM10 is necessary for proper ectodomain shedding of ICOSL. Impaired ICOSL shedding leads to accumulation of B cell surface ICOSL which stimulates internalization of ICOS on T cells. This excessive internalization results in inadequate ICOS signaling, impairing T follicular helper cell maturation, thereby reducing antibody production (23). In a murine model of SLE, ADAM10 deletion in B cells led to suppression of germinal center responses and reduced levels of antibodies directed against ds-DNA (111). This indicates, in contrast to the findings above, that inhibition of ADAM10 may be beneficial in the treatment of SLE.

Activation of macrophages in peripheral tissues is another significant component of SLE pathogenesis (106). Axl is a receptor tyrosine kinase found predominantly on macrophages which, upon binding of its ligand Gas6, inhibits production of pro-inflammatory cytokines (112). ADAM10 promotes shedding of Axl, leading to hyperactivity of tissue macrophages and exacerbation of tissue damage in a murine model of lupus (113). Significantly, the cleaved, soluble form of Axl was found to be increased in serum from patients with active SLE flares compared to SLE patients without active disease and healthy controls (113). This, like the findings of Hoffman et al. indicate that activation of ADAM10 may ameliorate SLE symptoms and slow disease progression.

These studies indicate that ADAM10 may be a relevant therapeutic target in SLE. To date, however, the evidence for modulating ADAM10 in SLE remains unclear. It seems that inhibition of ADAM10 on B cells would reduce TACI cleavage, leading to increased T-independent B cell activation, but may also alter ICOS-ICOSL signaling leading to excessive antibody production. Further study is needed to elucidate the implications of targeting ADAM10 in SLE as a therapeutic strategy. It is likely that the role of ADAM10 in SLE is highly spatio-temporally dependent and the effects of modulating ADAM10 may vary greatly depending upon when intervention occurs in the natural history of the disease.

### Psoriasis

Psoriasis is an inflammatory disease that is characterized by hyperproliferative lesions of the skin that are associated with immunological dysregulation and aberrant keratinocyte differentiation (114). Keratinocyte differentiation is critically regulated by Notch signaling which drives sequential maturation from basal stem cells to spinous layer keratinocytes (115, 116). In Notch signaling, Notch ligand binding induces a conformational change in Notch, exposing the negative regulatory region for cleavage by ADAM10. This cleavage event provides a substrate for γ-secretase to perform an additional cleavage to generate the transcriptionally active NICD (17). Epithelial deletion of ADAM10 in adult mice results in hyperproliferation of keratinocytes and dysregulated keratinocyte differentiation due to impaired Notch signaling. Lesions from these mice resemble the architecture of human psoriatic lesions, exemplified by a thickened epidermis and proliferative basal-like cells present in suprabasal epidermal layers (117).

It remains unclear whether psoriatic lesions display derangements in ADAM10 expression or activity. One study demonstrated upregulation of ADAM10 in keratinocytes in psoriatic lesions, with increased levels in deeper layers of the epidermis compared to healthy controls (118). These findings, however, are based on only a single immunohistochemistry study. Thus, further study of the role of ADAM10 in keratinocyte differentiation and propagation of psoriatic inflammation is needed.

Clinically, acitretin is an oral retinoid that is approved for psoriasis treatment (119). Acitretin functions as a retinoic acid receptor agonist that promotes keratinocyte differentiation. Activation of retinoic acid receptors, however, also induces expression of ADAM10 *in vitro* (120). The upregulation of ADAM10 by acitretin may facilitate Notch signaling in keratinocytes, thereby restoring normal keratinocyte differentiation and epidermal architecture. Tamibarotene, cilostazol, and resveratrol are additional agents that induce the expression of ADAM10 via activation of retinoic acid receptors (121, 122). The effects of these drugs may be comparable to acitretin in the treatment of psoriasis and warrant further study to investigate their efficacy and side effect profiles.

Currently, direct small molecule activators of ADAM10 are not available. The ADAM10 activators that have been described are etazolate, bryostatin, and (–)-epigallocatechin-3-gallate (EGCG) (123). These drugs, however, activate ADAM10 through a mediating receptor. Etazolate activates ADAM10 secondary to activation of the GABAA receptor and therefore displays significant tropism to cells of the central nervous system (124). Bryostatin activates ADAM10 via protein kinase C and can therefore induce ADAM10 activity in a variety of cell types (125, 126). EGCG, a natural occurring compound derived from green tea, has also been described to induce ADAM10 activity (127). At present, the target receptor for EGCG remains unidentified. ADAM10 activation by EGCG, however, is clearly secondary to a tyrosine kinase, as tyrosine kinase inhibitors prevent EGCG induced ADAM10 activation (127). Clinical trials for EGCG are underway and have demonstrated safety and tolerability in studies of prostate cancer and Fragile X syndrome (128, 129). Considering the recent emergence of several biologics for treatment refractory psoriasis, these ADAM10 inducers may provide a valuable and cost-effective first line treatment option for psoriasis.

### Bullous Pemphigoid

Bullous pemphigoid (BP) is a severe autoimmune cutaneous blistering condition that is caused by autoantibodies directed against transmembrane collagen XVII (anti-BP180). These antibodies interfere with epidermal basal cell adhesion, leading to separation of the dermis from the epidermis which causes blistering (130). The central role of pathological B cell activation in BP was illustrated in a study by Hall et al. in which patients with recalcitrant disease exhibited a significant reduction in disease activity following B cell depletion by rituximab (131). Analysis of serum and blister fluid revealed elevated levels of semaphorin 4D, which augments production of BP180 antibodies *in vitro*. Semaphorin 4D release is derived from CD15^+^ granulocytes and occurred through an ADAM10-dependent mechanism (132). These findings indicate that inhibition of ADAM10 could suppress autoreactive antibody production in BP, thereby reducing disease activity.

## Conclusions

The overwhelming data in cancer and autoimmunity, even added within the last 5 years implicates ADAM10 in the progression of disease. The popularity of ADAM inhibition in the 90s is returning, and the larger question is, will it be merely a passing trend? Early, the need for compounds that were specific for ADAM10 was made especially evident following the failure of the first generation metalloproteinase inhibitors, Batimastat, Marimastat, and Neovastat early in clinical trials due to reports of adverse side effects (133, 134) Even with the addition of specific ADAM10 targeting drugs, the other major obstacle was treating ADAM10 at the point of therapeutic intervention. With the ubiquitous nature of ADAM10 throughout human cell types and tissues, this has been extremely difficult. Unless ADAM10 intervention is accompanied by a drug delivery system that allows for a targeted approach, there is almost certainly going to be off-target and on-target effects (through inhibition of Notch cleavage) leading to adverse events.

Even in the absence of a cell- or tissue-specific approach of targeting ADAM10, potential still exists in the right clinical setting. New inhibitor design may also improve off-target and on-target effects, as well as be able to generate a more selective inhibitor between ADAM10 and ADAM17. Recent structural work from Seegar et al. may yield improved inhibitors of ADAM10, as more is now known about the structure and auto-modulation through the disintegrin cysteine-rich domain (5). This, paired with improved knowledge of tetraspanin regulation of ADAM10, may allow generation of better inhibitors that are able to specifically target ADAM10's cleavage of particular substrates (6). Further, protease inhibitors may be surpassed in specificity through the use of monoclonal antibodies that mask the binding pocket for ADAM10, like mAb 8C7 (135). Yet, without the implementation of a personalized medicine approach, especially in cancer, ADAM10 inhibition therapy will most likely continue to fail in clinical trials. Identifying patients who will benefit the most from ADAM10 inhibition therapy should be the priority, as well as applying ADAM10 inhibitors to prevent resistance of the tumor to standard treatments such as anti-CD30 or anti-HER2. In autoimmunity the story remains less clear, yet most autoimmune diseases have relevant targets.

Multiple reviews have been published supporting the potential of ADAM10 protein in platelets and cerebrospinal fluid to serve as a biomarker for Alzheimer's disease diagnoses (82, 136, 137). A study that examined in the urinary vesicles of patients with glomerular kidney diseases found higher levels of ADAM10 (138). ADAM10 is also overexpressed in the synovial tissue of RA patients (95). It has been reported that ADAM10 is correlated with disease activity and regulates monocyte migration and adhesion in RA patient fluids (96, 97). ADAM10 elevation in specific cells or tissues correlates strongly with various disease states. However, ADAM10 is also elevated broadly in many cancers on exosomes. Exosomal ADAM10 was found to be elevated in plasma of cancer patients, and was thought to be an additional pan-cancer marker (90). The use of ADAM10 expression in plasma or ADAM10 substrates as biomarkers in cancer or for disease progression are extremely promising. But, the studies in this review, aside from Hsp90α, need replication in multi-center validation studies prior to use in a clinical setting. The additional complication is the ubiquitous nature of ADAM10 expression and the upregulation in multiple disease states could cause issues with its use as a biomarker in situations with comorbidities.

Overall, ADAM10 is still extremely targetable. Improvements in drug delivery, reduction in off-target effects, and careful identification of patient populations will be needed to successfully move these drugs into the clinic.

## Author Contributions

TS, AT, and RM all participated in the writing, editing, and overall construction of this review.

### Conflict of Interest

The authors declare that the research was conducted in the absence of any commercial or financial relationships that could be construed as a potential conflict of interest.
